# Nourish to Flourish: A Mindful Eating Program’s Impact on Health and Wellbeing Among Older Adults

**DOI:** 10.1093/geroni/igaf122.3247

**Published:** 2025-12-31

**Authors:** Ajla Basic, Teresa Fung, Catherine O’Brien

**Affiliations:** Mather Institute, Evanston, Illinois, United States; Harvard T.H. Chan School of Public Health, Boston, Massachusetts, United States; Mather Institute, Evanston, Illinois, United States

## Abstract

Mindful eating is an evidence-based approach that enhances awareness of food choices, eating behaviors, and overall well-being. This study explores outcomes of Nourish to Flourish, a four-week mindful eating program designed for adults 50+ across seven sites in the Chicagoland area from July to October 2024. Facilitators guided participants through a structured curriculum that emphasized eating with intention, recognizing hunger and fullness cues, and fostering a positive relationship with food. Participants completed pre- and post-program surveys assessing mindful eating behaviors, self-reported health, spirituality, and engagement in healthy behaviors. Among 98 participants who attended the first session, 78 (89.6% females) completed the pre and post survey. Findings revealed statistically significant improvements from pre to post in the following measures: mindful eating (M=-.416; SD=.527); [t(77) = -6.97, p <.001], healthy eating identity (M=-.319; SD=.755) [t(73) = -3.64, p < .001] and spirituality (M=-1.08; SD = 2.62); [t(70) = -3.47, p < .001] Statistically improvement in self-reported health was also observed (M=-.197; SD=.748); [t(75) = -2.29, p < .024]. Additionally, participants evaluated the program highly; 93.4% rated their satisfaction with the program highly and 100% claimed they would continue mindful eating practices. In conclusion, a 4-week mindful eating program was successful in improving a number of aspects in dietary and holistic health. Future studies may focus on the long term sustainability of these results.

